# Social cognitive theory mediators of physical activity in a lifestyle program for cancer survivors and carers: findings from the ENRICH randomized controlled trial

**DOI:** 10.1186/s12966-016-0372-z

**Published:** 2016-04-14

**Authors:** F. G. Stacey, E. L. James, K. Chapman, D. R. Lubans

**Affiliations:** School of Medicine and Public Health, The University of Newcastle, Hunter Medical Research Institute, Priority Research Centre for Health Behavior, Priority Research Centre in Physical Activity and Nutrition, Level 4 West, HMRI Building, Callaghan, NSW 2308 Australia; Cancer Council New South Wales, 153 Dowling St, Woolloomooloo, NSW Australia; School of Education, and Priority Research Centre in Physical Activity and Nutrition, The University of Newcastle, ATC Building, Callaghan, NSW 2308 Australia

**Keywords:** Physical activity, Cancer, Mediators, Social cognitive theory

## Abstract

**Background:**

Despite increasing numbers of cancer survivors and evidence that diet and physical activity improves the health of cancer survivors, most do not meet guidelines. Some social cognitive theory (SCT)-based interventions have increased physical activity behavior, however few have used objective physical activity measures. The Exercise and Nutrition Routine Improving Cancer Health (ENRICH) randomized controlled trial reported a significant intervention effect for the primary outcome of pedometer-assessed step counts at post-test (8-weeks) and follow-up (20-weeks). The aim of this study was to test whether the SCT constructs operationalized in the ENRICH intervention were mediators of physical activity behavior change.

**Methods:**

Randomized controlled trial with 174 cancer survivors and carers assessed at baseline, post-test (8-weeks), and follow-up (20-weeks). Participants were randomized to the ENRICH six session face-to-face healthy lifestyle program, or to a wait-list control. Hypothesized SCT mediators of physical activity behavior change (self-efficacy, behavioral goal, outcome expectations, impediments, and social expectations) were assessed using valid and reliable scales. Mediation was assessed using the Preacher and Hayes SPSS INDIRECT macro.

**Results:**

At eight weeks, there was a significant intervention effect on behavioral goal (A = 9.12, *p* = 0.031) and outcome expectations (A = 0.25, *p* = 0.042). At 20 weeks, the intervention had a significant effect on self-efficacy (A = 0.31, *p* = 0.049) and behavioral goal (A = 13.15, *p* = 0.011). Only changes in social support were significantly associated with changes in step counts at eight weeks (B = 633.81, *p* = 0.023). Behavioral goal was the only SCT construct that had a significant mediating effect on step counts, and explained 22 % of the intervention effect at 20 weeks (AB = 397.9, 95 % CI 81.5–1025.5).

**Conclusions:**

SCT constructs had limited impact on objectively-assessed step counts in a multiple health behavior change intervention for cancer survivors and their carers. Behavioral goal measured post-intervention was a significant mediator of pedometer-assessed step counts at 3-months after intervention completion, and explained 22 % of the intervention effect. Future research should examine the separate impact of goals and planning, as well as examining mediators of behavior maintenance in physical activity interventions targeting cancer survivors.

**Trial registration:**

Australian and New Zealand Clinical Trials registry ANZCTRN1260901086257.

## Background

The number of cancer survivors is increasing due to the aging population and improvements in early detection and cancer treatments [1]. There are an estimated 28 million people worldwide living with cancer who were diagnosed in the previous five years [2]. Cancer survivors are at-risk of secondary cancers, other co-morbidities (like diabetes and cardiovascular disease), and poor physical and psychosocial health [3]. Current guidelines for cancer survivors report that physical activity (PA) can be safely performed both during and after cancer treatment [4–6], and PA has been shown to improve survival, risk of recurrence and side-effects from cancer and its treatments [7–13]. Despite these benefits, it is estimated that only 28 to 47 % of cancer survivors meet PA guidelines [14–16]. To date, there have been a number of trials investigating the efficacy of PA in clinical settings. However, there have been fewer trials investigating how to promote long-term behavior change in cancer survivors. Most PA trials have targeted breast cancer survivors and have focussed on short-term outcomes (12 weeks) [17–19]. Few PA trials have used an objective PA measure, or assessed the impact of behavior change after the intervention [17–19]. Carers of cancer survivors share many of the same behavioral risk factors as survivors [20, 21], and also experience poor physical and psychosocial health [22], however they are rarely targeted in interventions.

Interventions that are based on theory have shown promise in promoting positive behavior change [23–25]. Theory-based interventions allow for an exploration of why an intervention worked, and what strategies were crucial to their success [26–28]. While many interventions claim to be theory-based, often their theoretical constructs are inadequately described, and the constructs rarely tested [25, 29–31]. Mediation analysis can be used to identify the most effective components of an intervention and help to explore the mechanisms of behavior change. A previous review of PA interventions in adult non-clinical populations reported that only half of reviewed studies showed evidence that the intervention changed PA and the proposed mediator of behavior change, and the outcomes of mediation were mixed [32]. Identifying the mechanisms of behavior change is important in refining existing theories, and developing new theories of behavior change.

Social cognitive theory (SCT) offers principles on how to predict and change health behavior [33]. Knowledge of health risks and benefits precede all the SCT constructs, with self-efficacy affecting behavior directly, and indirectly by the impact on goals, outcome expectations, and perceived facilitators and impediments [33]. The category of ‘goals’ is broad, and for the purpose of this paper, the term goal or behavioral goal may be defined as “detailed planning of what the person will do, including definition of the behavior specifying frequency, intensity or duration” [26], and includes both proximal and distal goals [34]. The outcome expectations are the perceived positive and negative effects of the behavior, which are directly influenced by self-efficacy [33, 35]. Self-efficacy includes confidence to overcome barriers to successful behavior change, as well as the ability to perform and assess behavior under a range of personal, social, and environmental conditions [33]. In a systematic review and meta-analysis (*n* = 12 trials) of SCT-based interventions for cancer survivors, SCT-based interventions were found to be effective at changing PA behavior with an effect size of 0.33 [25]. However, the studies were heterogeneous and there were no specific SCT constructs, or specific intervention delivery modes that were related to intervention efficacy, and results did not differ if the intervention targeted single or multiple health behavior interventions [25].

The Exercise and Nutrition Routine Improving Cancer Health (ENRICH) trial is a theoretically-based multiple health behavior change intervention for cancer survivors and carers. ENRICH was evaluated using a randomized controlled trial and reported significant intervention effects on mean daily step counts (as measured by 7 days of pedometry) at both 8- and 20-week follow-up [36]. The intervention, based on SCT [33] and a chronic disease self-management model [37], consisted of healthy eating knowledge and skill development, resistance training principles and exercises utilizing a Gymstick™ and a home-based walking program using a pedometer. The aim of the current study was to investigate whether the SCT constructs targeted in the intervention served as mediators of the intervention effect on pedometer-assessed step counts at 8- and 20-weeks.

## Methods

### Study design

The methods and primary analysis have been described in detail elsewhere [36, 38]. In brief, people with a previous diagnosis of cancer and their carers were recruited from health professionals, cancer support groups, media, and support services of a cancer charity in Sydney, Australia, during 2010 to 2012. The reporting of the trial conformed to the Consolidated Standards of Reporting Trials (CONSORT) guidelines for pragmatic RCTs [39]. The trial was registered with the Australian and New Zealand Clinical Trials registry (ANZCTRN1260901086257), and ethics approval was obtained from the University of Newcastle Human Research Ethics Committee (H-2009-0347).

### Sample

Included participants were: 1) individual diagnosed with cancer who had completed all active cancer treatment (“cancer survivor”), or carer of cancer survivor; 2) no food or dietary restrictions as a result of surgery or treatment; 3) aged 18 years or older; 4) fluent in English; 5) signed medical clearance from their General Practitioner; and 6) with a functional performance score of two or less on the Eastern Cooperative Oncology Group criteria (that is “at least ambulatory and capable of all self-care…or up and about more than 50 % of waking hours”) [40]. Participants provided informed consent.

### Intervention

The ENRICH program involved six face-to-face group education and skill development sessions held over 8 weeks. Participants were provided with a workbook (which contained program notes, activities, and handouts), an open pedometer and a Gymstick™ (a lightweight graphite shaft, with elastic tubing and foot straps that provide resistance to exercise all major muscle groups) [41]. Each group-based session delivered simultaneous multiple health behavior content covering a home-based walking program (using a pedometer), home-based resistance training program (using a Gymstick™), and information about healthy eating (the Australian Guide to Healthy Eating, fruit and vegetables, maintaining a healthy weight, fats, meat, salt, dietary supplements, alcohol, and reading food labels). Sessions included a mix of didactic information delivery and practical activities. Each session was co-facilitated by a qualified exercise specialist (Accredited Exercise Physiologist or Physiotherapist) and an Accredited Practising Dietician. The content and delivery of sessions was operationalized using the principles of SCT [33] and a chronic disease self-management framework [37]. The specific theoretical constructs that were operationalized included knowledge, behavioral goals, self-efficacy, outcome expectations, impediments, and social support (see Table 1). Whilst ENRICH was a multiple health behavior change intervention targeting both PA (aerobic and resistance) and healthy eating, the focus of this paper is on the primary outcome of pedometer-assessed step counts. Mediators of dietary change were not assessed, due to the complexity and number of individual dietary behaviors that were targeted in the intervention (fruit, vegetables, fat, salt, meat consumption, energy, alcohol), and the lack of brief, validated measures. The specific intervention strategies, how they relate to each theoretical construct, and the specific behavior change techniques from the CALO-RE taxonomy [42] are detailed in Table 1.

**Table 1:** Description and psychometric properties of hypothesized mediator scales

Construct	Intervention strategies	Behavior change techniques from the CALO-RE taxonomy [42]	Description of scale	Range; number of items	Sources	Cronbach alpha α
Behavioral goal	• Plan home walking program with step count goals • Revise step count goals • Set goal to reduce sitting time • Goals about how often to walk • How to revise and set new goals • Resistance training behavior contract • How to stay on track after ENRICH by setting goals	BCT#5-Goal setting (behavior); BCT#6-Goal setting (outcome); BCT#7–Action planning; BCT#10–prompt review of behavioral goals; BCT #11-prompt review of outcome goals.	Scale from 0-100 %	0–100 %; 1 item	Courneya, et al., 2000 [75]	N/A
			Participants were asked to indicate “How likely is it that you will do regular PA within the next eight weeks?”			
Self-efficacy	• Resistance training handbook – goal setting and self-monitoring • Know how to use Gymstick™ and participated in resistance training session • How to modify resistance training programs as fitness improves • Learn new Gymstick™ exercises • Review resistance training progress • Participated in resistance training fitness circuit • How to plan a home walking program • Strategies to increase exercise adherence • Devised personal home walking program to carry out upon ENRICH completion • Tips to keep motivated	BCT#16–prompt self-monitoring of behavior; BCT#17-prompt self-monitoring of behavioral outcome; BCT#21–provide instruction on how to perform the behavior; BCT#22-model/demonstrate the behavior; BCT#26–prompt practice; BCT#27–use of follow-up prompts.	5-point Likert format: 1 = not at all confident to 5 = extremely confident. Participants were asked to rate their confidence that they could participate in regular PA over the next eight weeks when: Eg. When I am in a bad mood or feeling depressed….	1–5; 9 items	Plotnikoff, et al., 2001 [76]	0.90
Outcome expectations	• Familiar with Rate of Perceived Exertion (RPE) scale • Participated in resistance training fitness circuit • Group discussion on resistance training progress and training adherence • Use testing and assessment for motivation and chart improvement • Record your activity and thoughts before during and after exercise to help improve adherence and barriers • Use training diaries to record PA and exercise sessions	BCT#16–prompt self-monitoring of behavior; BCT#17–prompt self-monitoring of behavioral outcome; BCT#23–teach to use prompts/cues; BCT#24–environmental restructuring; BCT#28–facilitate social comparison; BCT#29–plan social support/social change; BCT#31–prompt anticipated regret; BCT#35–relapse prevention/coping planning.	5 point Likert scale: 1 = strongly disagree to 5 = strongly agree. Participants were asked to select how much they agree with the 5 statements that participating in regular PA over the next eight weeks would for them: Eg. Reduce tension or manage stress	1–5; 5 items	Plotnikoff, et al., 2001 [76]	0.91
Impediments	• Reflect on diaries and identify solutions to barriers • Strategies to increase exercise adherence • What are the barriers people are facing? • Resistance training behavior contract	BCT#8–barrier identification/problem solving; BCT#18–prompting focus on past success.	5 point Likert scale: 1 = strongly disagree to 5 = strongly agree. Participants were asked to select how much they agree with the 5 statements that participating in regular PA over the next eight weeks would for them: Eg. Take too much of my time.	1–5; 5 items	Plotnikoff, et al., 2001 [76]	0.72
Social support	• Inclusion of partners/carers in attending ENRICH program • Group discussion and brainstorming • Face-to-face group sessions • Encouraged to use social support and to do PA together (to keep motivated)	BCT#29–plan social support/social change.	5 point Likert format: 1 = Not at all to 5 = very much. Participants are asked whether over the next eight weeks people in their social network are likely to help them participate in regular PA, and whether they feel that someone in their social network will provide the support they need in order to be regularly physically active.	1–5; 2 items	Courneya, et al., 2000 [75]	0.91

Control: After completing 20-week study measures, control group participants (*n* = 58) attended the ENRICH program.

### Assessments

Data were collected by written survey, wearing a sealed pedometer, and completing a concurrent log sheet, at baseline, eight weeks (intervention completion), and 20-weeks (3 months post-intervention, 5 months post-randomization).

The primary outcome was step counts, as measured by sealed pedometer (Yamax SW200) for seven days. Participants recorded time worn, and occasions of other activities (resistance training, swimming, water aerobics, cycling) not captured by pedometry. These activities were converted to sex-specific step counts using previously reported values [43] and were added to the total step count value.

A description and psychometric properties of each of the hypothesized mediators assessed by written survey is reported in Table 1. The hypothesized mediators included behavioral goal, self-efficacy, outcome expectations, impediments, and social support. An eight week time reference, and definition of ‘regular PA’ was provided for the hypothesized mediators. Consistent with PA guidelines, ‘regular physical activity’ was defined as “achieving at least 30 minutes of moderate or vigorous-intensity activity on most, preferably all, days of the week” [44].

### Statistical analyses

**Fig. 1:**
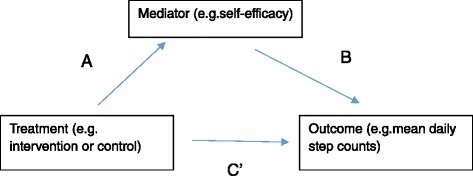
Mediation analysis overview. **a** = Action theory. **b** = Conceptual theory. **c**  = Direct effect

Analyses were performed using IBM SPSS Statistics for Windows, Version 21 (IBM Corp, Armonk, New York, USA). Relevant items were reverse-coded, and a mean score for each construct was computed. Cronbach alphas for each construct, except for behavioral goal, are reported in Table 1. Mediation analyses were conducted using the INDIRECT macro developed by Preacher and Hayes [45]. The macro computes the following steps simultaneously: i) regression coefficients for the impact of the intervention on the potential mediators (pathway A or action theory); ii) the associations between changes in mediators and changes in pedometer-assessed step counts, independent of study group allocation (pathway B or conceptual theory); iii) the total effects (pathway C), direct effects (pathway C’), and indirect (pathway AB) intervention effects. The mediation pathways are illustrated in Fig. 1. Bias-corrected bootstrapped 95% asymmetrical confidence intervals were computed for the indirect effect [45]. Significant mediation was established if the confidence intervals did not include zero. The proportion of the intervention effect that was attributed to each mediator was computed by dividing the indirect effect (pathway AB) by the total effect (pathway C’ + pathway AB). Single mediator models were computed for pedometer-assessed step counts at 8- and 20-weeks, adjusting for baseline step count and mediator variables. Multiple mediator models were computed for step counts at 20-weeks, adjusted for baseline step count and mediator variables.

Mean scores of each SCT construct at eight weeks were used in all analyses. Mediation analysis was undertaken using a completers-only analysis, with sensitivity analysis to examine the impact of missing data. The estimation maximization algorithm in SPSS was used to impute missing outcome and mediator data. Pedometer-assessed data and Active Australia survey data were used to predict missing outcome data (pedometer-assessed step counts).

The result of Little’s Missing Completely At Random (MCAR) test confirmed that outcome data were missing completely at random (Chi-Square = 280.9, df = 282; *p* = 0.51). Missing mediator values were imputed for each individual item and the results of Little’s Missing Completely at Random (MCAR) test confirmed that mediator data were also missing completely at random. Results of the intention-to-treat mediation analysis were then compared to the completers-only analysis. Demographics of participants who completed the study (defined as not withdrawn at 20-weeks) were compared to those who dropped out of the study.

## Results

Participants (*n* = 174) were randomized and 133 completed baseline data collection [36]. At 8-week data collection, 82 % (*n* = 109) of the sample were retained, and at 20-weeks, 71 % (*n* = 94) of the sample were retained. The majority of participants who withdrew, did so prior to attending any ENRICH sessions (*n* = 51). Study groups had similar baseline demographic characteristics (see Table 2). Three-quarters of the sample were female, with mean age of 57 years, and were cancer survivors. The majority of cancer survivors were diagnosed with breast cancer, and had been diagnosed three to four years previously. There were 24 carers in the sample and 12 participants who were both cancer survivors and carers.

**Table 2:** Baseline characteristics of participants (*n* = 133)

	Control (*n* = 58)		Intervention (*n* = 75)	
Characteristic	N	%	N	%
Age, years, Mean (SD)	58.1 (11.2)		56.2 (12.6)	
Female gender	43	74.1	60	80.0
Married/de facto	38	66.7	55	73.3
Completed post-school qualifications	41	71.9	54	73.0
Employed (full-time or part-time)	26	45.6	34	45.9
Weekly family income				
-Less than $499	11	19.6	12	16.0
-$500-$1000	14	25.0	16	21.3
-More than $1000	14	25.0	25	33.3
-Prefer not to answer	17	30.4	22	29.3
Cancer survivor status				
-Cancer survivor	43	74.1	53	70.7
-Carer	9	15.5	15	20.0
-Both cancer survivor and carer	5	8.6	7	9.3
Relationship to cancer survivor:				
-Spouse/partner	11	78.6	12	54.5
Cancer type^a^				
-Breast	28	58.3	36	60.0
-Prostate	7	14.6	7	11.7
-Other: *colorectal, melanoma, non-Hodgkins lymphoma, leukaemia, ovarian, thyroid.*	21	43.8	24	40.0
Time since diagnosis, months, Mean (SD)	45.2 (52.3) or 3.7 years		39.3 (56.7) or 3.2 years	
Ever received the following cancer treatments^a^				
-Surgery	45	93.8	55	93.2
-Chemotherapy	28	62.2	45	84.9
-Radiotherapy	30	63.8	32	68.1
-Hormone treatment	20	48.8	30	66.7
Cancer in remission	36	80.0	44	77.2

^a^Participants could select more than one response, so the percentage may add up to more than 100 %

A comparison of participants who dropped out, and those who completed the study is reported in Table 3. Compared to those who completed the study, people who dropped out of the study were significantly more likely to be in the intervention group (73 % vs 49 %; *p* = 0.009), and to report being diagnosed with arthritis (54 % vs 32 %; *p* = 0.028). Participants who dropped out of the study were also more likely to report co-morbidities (88 % vs 74 %), mental health problems (44 % vs 28 %), to report longer time since diagnosis (4.2 years vs 3.2 years), and were more likely to have received chemotherapy (83 % vs 71 %), however these differences did not reach statistical significance. However, there were no differences in the mediation results between the completers analysis and the intention-to-treat analysis. Therefore, the results of the completers analysis was reported as the primary mediation analysis with the intention-to-treat reported as a sensitivity analysis.

**Table 3:** Demographic comparison of participants who completed the study and participants who withdrew prior to 20-week data collection

	Completers (*n* = 92)		Dropouts (*n* = 41)		*P*-value
Characteristic	N	%	N	%	
Study group					0.009*
-Intervention	45	48.9	30	73.2	
-Control	47	51.1	11	26.8	
Age, years, Mean (SD)	57.0 (12.0)		57.1 (12.1)		0.960
Female gender	72	78.3	31	75.6	0.736
Married/de facto	67	72.8	26	65.0	0.365
Completed post-school qualifications	64	69.6	31	79.5	0.245
Employed (full-time or part-time)	40	43.5	17	43.6	0.991
Weekly family income					0.178
-Less than $499	12	13.2	11	27.5	
-$500–$1000	24	26.4	6	15.0	
-More than $1000	28	30.8	11	27.5	
-Prefer not to answer	27	29.7	12	30.0	
Number of co-morbidities (ever or current)					0.073
-0	24	26.1	5	12.2	
-1 or more	68	73.9	36	87.8	
Types of co-morbidities^a^					
-Musculoskeletal disorders	33	36.3	15	38.5	0.834
-Mental health problems	25	27.5	17	43.6	0.086
-Arthritis	29	31.9	21	53.8	0.028*
-High blood pressure	24	26.4	12	30.8	0.608
-High cholesterol	33	36.3	11	28.2	0.396
Cancer survivor status					0.535
-Cancer survivor	65	70.7	31	77.5	
-Carer	19	20.7	5	12.5	
-Both cancer survivor and carer	8	8.7	4	10.0	
Relationship to cancer survivor:					
-Spouse/partner	17	63.0	6	66.7	0.397
Cancer type^a^					
-Breast	47	64.4	17	48.6	0.118
-Prostate	8	11.0	6	17.1	0.375
-Other: *colorectal, melanoma, non-Hodgkins lymphoma, Leukaemia, ovarian, thyroid, lung.*	17	23.2	7	20.0	
Time since diagnosis, months, Mean (SD)	38.0 (43.5) or 3.2 years		50.1 (72.3) or 4.2 years		0.293
Ever received the following cancer treatments^a^					
-Surgery	69	94.5	31	91.2	0.677
-Chemotherapy	49	71.0	24	82.8	0.431
-Radiotherapy	42	64.6	20	69.0	0.759
-Hormone treatment	35	70.0	15	62.5	0.632
Cancer in remission	54	77.1	26	81.3	0.356
BMI category (kg/m^2^)					0.468
-Less than 25	31	34.4	16	43.2	
-25–30	36	40.0	14	37.8	
-30 and above	23	25.6	7	18.9	

*denotes significant difference (*P* < 0.05)

^a^Participants could select more than one response, so the percentage may add up to more than 100 %

### Intervention effects

Overall intervention effects have been reported elsewhere [36]. In summary, significant group-by-time effects were found for mean daily steps at 8-weeks (adjusted mean difference 2810 steps/day; 95 % CI 1238–4382) and at 20-weeks (adjusted mean difference 2782 steps/day, 95 % CI 818–4745) (*P* = 0.0009) (see Table 4). Mean values for mediators at all three time-points are also reported in Table 4.

**Table 4:** Participants’ values for pedometer-assessed step count and hypothesized mediators

	Intervention (*n* = 75)			Control (*n* = 58)		
	Mean (SD)			Mean (SD)		
Model variable	Baseline	8 weeks	20 weeks	Baseline	8 weeks	20 weeks
Mean daily steps	8815 (3778)	10849 (5127)*	10307 (4446)*	9604 (5471)	8014 (4568)*	8026 (4698)*
Behavioral goal (0–100%)^a^	69.6 (29.7)	76.3 (24.3)	72.4 (24.5)	65.1 (32.7)	64.3 (32.4)	70.2 (27.2)
Self-efficacy (1–5)^a^	3.2 (0.8)	3.3 (0.9)	3.2 (0.9)	3.1 (0.7)	3.1 (0.9)	3.2 (0.9)
Outcome expectations (1–5)^a^	4.2 (0.8)	4.3 (0.6)	4.2 (0.7)	4.2 (0.8)	4.0 (0.9)	4.1 (0.9)
Impediments (1–5)^a^	3.7 (0.8)	3.9 (0.6)	3.8 (0.7)	3.9 (0.7)	3.8 (0.7)	3.9 (0.7)
Social support (1–5)^a^	3.0 (1.4)	2.8 (1.4)	2.7 (1.4)	2.8 (1.4)	2.6 (1.3)	2.8 (1.2)

*denotes significant difference (*P* < 0.05) using t-test to test for differences between study groups at each time-point, in relation to baseline

^a^denotes raw mean score; does not include imputation for missing data

### Mediation effects

The results of the mediation analysis are reported in Table 5.

**Table 5:** Action theory test, conceptual theory test and significance of the mediated effect on pedometer-assessed step counts

		Action theory		Conceptual theory		Direct effect		Indirect effect		
Hypothesized mediators	Time (weeks)	A (SE)	*p*-value	B (SE)	*p*-value	C’ (SE)	*p*-value	AB (SE)	95 % CI	AB/(C’ + AB) [Proportion (%)
Self-efficacy^a^	8	0.22 (0.13)	0.08	−66.86 (498.07)	0.89	1944.66 (588.37)	0.001*	−14.86 (107.57)	−300.97 to 157.42	−0.01 (−1%)
Self-efficacy^b^	20	0.31 (0.15)	0.05*	170.93 (608.68)	0.78	1652.18 (797.21)	0.042*	52.15 (166.17)	−200.98 to 514.51	0.03 (3%)
Behavioral goal^c^	8	9.12 (4.14)	0.03*	5.44 (15.93)	0.73	1873.05 (611.42)	0.003*	49.56 (133.97)	−170.91 to 382.83	0.03 (3%)
Behavioral goal^d^	20	13.15 (5.01)	0.01*	30.26 (19.19)	0.12	1396.80 (826.62)	0.10	397.88 (219.14)	81.50 to 1025.48*	0.22 (22%)
Outcome expectations^a^	8	0.25 (0.12)	0.04*	−168.47 (528.22)	0.75	1981.44 (591.81)	0.001*	−41.42 (93.61)	−343.99 to 80.66	−0.02 (−2%)
Outcome expectations^b^	20	0.24 (0.14)	0.09	659.94 (664.96)	0.32	1545.46 (786.64)	0.05*	157.74 (141.27)	−35.94 to 563.70	0.09 (9%)
Impediments^a^	8	0.14 (0.11)	0.21	341.97 (572.86)	0.55	1870.59 (585.45)	0.002*	47.88 (93.65)	−66.83 to 361.66	0.03 (3%)
Impediments^b^	20	0.16 (0.13)	0.22	−202.10 (726.23)	0.78	1655.60 (788.60)	0.04*	−32.32 (179.99)	−564.95 to 229.51	−0.02 (−2%)
Social support^a^	8	0.07 (0.22)	0.75	633.81 (274.49)	0.02*	1912.81 (561.93)	0.001*	45.53 (179.25)	−156.63 to 661.05	0.02 (2%)
Social support^b^	20	−0.05 (0.23)	0.82	578.01 (403.26)	0.16	1684.70 (761.42)	0.03*	−29.12 (157.80)	−492.06 to 235.47	−0.02 (−2%)
Multi-mediation (all)^e^	20	-	-	-	-	1499.81 (905.66)	0.10	118.48 (460.17)	−740.53 to 1292.25	7.32 (7%)

*denotes significant difference (*P* < 0.05)

^a^Sample size *n* = 88; ^b^Sample size *n* = 74; ^c^Sample size *n* = 85; ^d^Sample size *n* = 71; ^e^Adjusted for baseline steps and potential mediators at baseline

#### Action theory test

After controlling for baseline values, there were significant intervention effects for behavioral goal (A = 9.12, *p* = 0.031) and outcome expectations (A = 0.25, *p* = 0.042) at post-test (8-weeks). At 20-weeks, there were significant intervention effects for self-efficacy (A = 0.31, *p* = 0.049) and behavioral goal (A = 13.15, *p* = 0.011).

#### Conceptual theory test

At 8-weeks, changes in social support were significantly associated with changes in pedometer-assessed step counts (B = 633.81, *p* = 0.023). At 20-weeks, there were no significant relationships between changes in any SCT constructs and pedometer-assessed step counts.

#### Significance of mediated effect

Changes in behavioral goal satisfied the criteria for mediation and explained 22 % of the intervention effect on pedometer-assessed step counts at 20-weeks (AB = 397.88, 95 % CI 81.5–1025.5). No other constructs had a significant mediation effect at eight or 20-weeks.

In a multiple mediator model that examined intervention effects at 20-weeks, the individual construct behavioral goal had a significant mediating effect on step counts (AB = 464.74, 95 % CI 25.9–1548.87), and in the model containing all of the SCT constructs, this model explained 7 % of the intervention effect.

Sensitivity analysis using the estimation maximization algorithm was undertaken to assess the impact of missing data from those who did not complete the trial. There was no change in the main findings: behavioral goal remained a significant construct at 8- and 20-weeks, and explained 10 % of the intervention effect on step counts (AB = 186.2; 95 % CI 13.6–606.9).

## Discussion

The purpose of this study was to identify if constructs from SCT mediated changes in pedometer-assessed step counts in the ENRICH intervention for cancer survivors and carers. This study demonstrated that behavioral goal mediated the effect of the ENRICH intervention on step counts at 20-week follow-up, and accounted for 22 % of the intervention effect on step counts. No other constructs satisfied the criteria for mediation.

At post-test, the intervention was found to have a significant impact on behavioral goal and outcome expectations. There were no intervention effects for self-efficacy, impediments, or social support. At follow-up, the ENRICH intervention significantly improved self-efficacy and behavioral goal. However, behavioral goal was shown to have a significant mediating effect on pedometer-assessed step counts in both multiple and single mediator models, and explained between 7–22 % of the intervention effect. During the initial ENRICH session, participants set a goal to walk every day (at whatever time and distance was appropriate to their capability), and during subsequent sessions participants set step goals, monitored their steps using a pedometer and diary, reviewed and revised their goal each week. They were encouraged to write down SMART goals (specific, measurable, achievable, realistic, and with time-frames). Ninety-five percent of participants reported that ENRICH helped them to set reasonable goals that were within reach. This highlights the important role of goal setting and intentions in increasing PA behavior. Goal setting is a key element to many other behavior change theories, such as the Reasoned Action Approach [46] or Health Action Process Approach [47]. It may be that SCT is not the most appropriate behavior change theory for this target group and future research should examine the utility of other behavior change theories that hold goal setting as a central component. A meta-analysis of behavior change interventions has concluded that a medium-to-large change in intention leads to a small-to-medium change in behavior [48, 49]. Goals and intentions have been identified as a crucial part of behavior change and identified as a key part of theoretical frameworks, however there remains a gap between goal formation and behavior change. Recent literature has posited that planning is an important mediator between intentions and behavior [50, 51], and may help overcome the intention-behavior gap. The ENRICH trial did not assess the impact of goals and action planning separately, and future trials could incorporate goals and planning as distinct constructs, and test the causal pathway between goals, planning and behavior.

A review by Rhodes and colleagues examined the mediators of behavior change between selected SCT constructs and PA change and reported mixed results [32]. They also reported that, of the three trials that tested SCT constructs, none assessed or reported a conceptual theory link [32]; this is similar to the findings in this current analysis. However, in contrast to our results, other interventions based on SCT have reported that self-efficacy was the most commonly reported construct that influenced the results of the intervention [10, 52, 53]. Four studies reported improvements in self-efficacy were associated with increased PA [52–55]. However, mediation analyses in two trials identified that theoretical constructs only partially mediated intervention effects [56–58]. One SCT-based trial reported increased social support resulting from the intervention mediated the treatment effects on participants’ activity levels [54]. Similar to other studies, we found that outcome expectations and impediments were not mediators of PA [32, 54]. Ashford and colleagues reviewed 27 trials and found that half of the studies had included identification of PA barriers, and that this construct was significantly associated with lower self-efficacy [59]. There is limited support for SCT constructs as mediators of PA behavior change in this study and in other similar trials.

Response shift theory has been offered as an explanation for null findings in previous PA interventions targeting clinical populations [60, 61]. SCT posits that self-efficacy has direct influence on goals, outcome expectations, and impediments, as well as behavior [33]. Despite self-efficacy not being a significant mediator of change in this analysis, it may still have exerted important effects on other constructs, which are not accounted for in this analysis. Trials have supported links between self-regulatory efficacy and intentions [62]; self-efficacy and planning [50]; and intentions and barriers [48]. Behavior change techniques that prompt self-monitoring of behavioral outcomes and plan social support/social change have been associated with higher self-efficacy, and higher effect size for PA behavior change [63]. Measurement and analysis of the specific behavior change techniques was not undertaken in this study. Similar to other trials that used objective PA measures [54, 61, 64], changes in the SCT constructs explained a small amount of variance in PA. Alternatively, trials that have assessed PA by self-report have found that SCT constructs explain a greater amount of variance [65]. This is known as common method variance, and refers to the “variance that is attributable to the measurement method rather than to the constructs the measures are assumed to represent” [66]. Due to these differences, it is important to use objective measures of PA. As this was a highly motivated sample, it would be interesting to assess other constructs which may have been important, such as motivation, habit, or PA planning [32, 67, 68]. It could be that the constructs are different, depending on whether participants are trying to increase PA or maintain PA [67].

There were few differences between mediation analysis using the completers-only data or the intention-to-treat data. Using data from both eight and 20 weeks, the only construct shown to have a significant mediating relationship was behavioral goal which mediated the effect between the intervention and PA behavior change at 20 weeks, indicating that the completers-only analysis results are robust, despite the withdrawal rate.

### Limitations of this study

There was little change in the PA mediators as a result of the intervention, which raises several issues. The mediators were assessed in relation to “regular PA”, however the ENRICH intervention specifically targeted walking and resistance training. The lack of specificity may have also been an issue in how SCT constructs were defined. Self-efficacy was examined as one category, rather than breaking it down into the more specific constructs of task or barrier self-efficacy. There may have been cross-over or contamination between the individual construct measures, and it may be difficult to separate the individual effects of self-efficacy and outcome expectations [69]. The measure used to assess goal setting in this analysis is a measure of likelihood of performing regular PA, which may be a measure of motivation or intention, and makes it difficult to tease out separate effects of these constructs.

In addition, the mediator questions may not have been sensitive enough to detect change over eight weeks, particularly as baseline values were relatively high. It is not surprising that baseline values were high, as participants for this trial self-selected and were likely to be highly motivated to want to change PA and diet behaviors. Mediators of dietary change were not assessed, due to the complexity and number of individual dietary behaviors that were targeted in the intervention (fruit, vegetables, fat, salt, meat consumption, energy, alcohol), and the lack of brief, validated measures.

### Strengths of this study

The PA intervention was developed using SCT, with all PA constructs operationalized, and tested for their mediating effect. The rigorous application and testing of theoretical constructs is essential in moving theory forward and understanding the components crucial to intervention success. It is important to test for mediators in successful interventions to find out why they worked [70]. The ENRICH trial is a novel intervention that targeted survivors and carers of multiple cancer types, and focused on multiple lifestyle behaviors (PA/walking, resistance training, sitting, and a range of dietary behaviors). The inclusion of both cancer survivors and carers was expected to enhance social support. The lack of improvement in social support may have been due to the small number of carers who participated, or it could be that this strategy was not sufficient to improve participants’ perceived levels of social support. The success of the ENRICH intervention may be due to additional theoretical constructs (such as stage of change) or it may be related to specific behavior change techniques such as self-regulatory behaviors.

### Future research

Future studies should use a taxonomy of behavior change techniques to develop the ‘active ingredients’ of an intervention [71, 72]. Theoretical constructs have so far, shown mixed results in mediation analyses. There is some consistent evidence for specific behavior change constructs (eg self-regulatory behaviors) [54, 73, 74], and these specific behavior change constructs offer a promising way to develop potential future models of behavior change that should be tested in RCTs. Measures may need to be developed or refined to evaluate the change in these constructs. Use of an objective measure of behavior change outcome should be used in trials with PA or weight as an outcome. Maintenance of behavior change is an area that requires further intervention. We know little about how to support cancer survivors and carers to sustain positive behavior change. Identifying differences in mediators of behavior change and maintenance are important areas for future research.

## Conclusions

Behavioral goal was the only SCT construct to mediate the intervention effect on pedometer-assessed step counts at 20 week follow-up and accounted for 22 % of the intervention effect. The utility of behavioral goal for promoting PA was supported, however there was little evidence to support self-efficacy, outcome expectations, impediments, or social support for mediating the effect of PA behavior change. Future research should consider using the taxonomy of behavior change techniques to develop and evaluate interventions. It appears that the use of specific theoretical constructs and behavior change techniques offer the most promise for identifying the techniques critical to behavior change success.

## Abbreviations

BCT: behavior change technique
ENRICH: Exercise and Nutrition Routine Improving Cancer Health
PA: physical activity
RCT: randomized controlled trial
SCT: social cognitive theory
